# Laennec Attenuates Alcohol-Induced Hepatic Steatosis and Oxidative Stress in a Murine Model

**DOI:** 10.3390/cimb48070705

**Published:** 2026-07-10

**Authors:** Ju-Seop Kang, So-Jung Lim, Ryun Kang, So-Hyun Jeon, Chang-Taek Oh, Si-Young Jung, Sang-Hoon Lee

**Affiliations:** 1 Department of Pharmacology, College of Medicine, Hanyang University, Seoul 04736, Republic of Korea; 2 Department of Medical and Digital Engineering, College of Engineering, Hanyang University, Seoul 04736, Republic of Korea; 3 Research and Development Center, Green Cross Wellbeing Corporation, Yongin 16907, Republic of Korea; 4 Department of Family Medicine and Functional Medicine, Green Cross I-MED Gangnam Center, Seoul 06655, Republic of Korea

**Keywords:** Laennec, alcoholic liver disease (ALD), ALT, AST/ALT ratio, steatosis

## Abstract

Alcoholic liver disease (ALD) represents a major global health burden, encompassing a spectrum of hepatic abnormalities ranging from steatosis to cirrhosis and hepatocellular carcinoma. Laennec, a human placenta-derived hydrolysate, was evaluated for its therapeutic effects on alcohol-induced fatty liver in an experimental animal model. Animals were pretreated with alcohol for 2 weeks, followed by the co-administration of alcohol and Laennec for 4 weeks. The study included four groups: normal control, alcohol-only, and low- and high-dose Laennec-treated groups. Alcohol administration significantly elevated the serum ALT levels (3.17-fold vs. control), indicating hepatocellular injury, whereas Laennec treatment reduced the ALT levels in a dose-dependent manner. The AST levels increased in the alcohol-only group, although the change was not statistically significant; however, the AST levels decreased in the low-dose Laennec-treated groups. The AST/ALT ratio showed significantly dose-dependent recovery in the high-dose group. Laennec treatment attenuated hepatic lipid accumulation and inflammation, with the most pronounced effects observed in the high-dose group. Regarding alcohol-metabolizing enzymes, Laennec exhibited an inhibitory effect on alcohol-induced ADH activity, with no significant effect on ALDH. CYP2E1 activity was suppressed in a dose-dependent manner following Laennec administration. No significant changes were observed in the GPx activity. Additionally, high-dose Laennec significantly restored the catalase and STAT3 (Ser727) phosphorylation levels. Histopathological analysis (H&E staining) demonstrated marked reductions in lipid droplet accumulation and inflammatory cell infiltration in Laennec-treated groups compared to the alcohol-only group. These findings suggest that Laennec exerts hepatoprotective effects against alcohol-induced liver injury and steatosis, as evidenced by improvements in biochemical markers, enzyme activity, and histological features.

## 1. Introduction

Alcoholic liver disease (ALD) represents a major global health burden and encompasses a spectrum of hepatic abnormalities ranging from steatosis to cirrhosis and hepatocellular carcinoma. Chronic alcohol consumption induces hepatic lipid accumulation, oxidative stress, and inflammatory responses, ultimately leading to hepatocellular injury and disease progression [1]. Despite advances in understanding the underlying pathophysiology, effective pharmacological interventions capable of directly inhibiting disease progression remain limited, and long-term abstinence continues to be the most reliable strategy for improving the clinical outcomes.

Laennec, a human placental hydrolysate (HPH), is widely used as a therapeutic agent for improving liver function in chronic liver disease [2]. Human placental hydrolysates are produced through enzymatic and chemical hydrolysis processes, yielding low- and medium-molecular-weight bioactive compounds [3,4]. These preparations contain various biologically active components, including cytokines, growth factors (e.g., hepatocyte, epidermal, and insulin-like), amino acids, vitamins, and nucleic acid derivatives [4,5]. Such components have been reported to exert antioxidant, anti-inflammatory, and regenerative effects, including the activation of hepatic antioxidant enzymes such as superoxide dismutase, catalase, and glutathione peroxidase, as well as the promotion of hepatocyte proliferation and functional recovery.

The therapeutic application of placental-derived products has evolved from crude extracts to standardized hydrolysate formulations with improved safety, reproducibility, and bioavailability [6]. These formulations have been clinically utilized in Japan since the mid-20th century and are currently approved in South Korea for the treatment of chronic liver dysfunction [2]. Accumulating evidence indicates that human placental hydrolysates exert hepatoprotective effects through multiple mechanisms, including the enhancement of detoxification pathways, the attenuation of oxidative stress, and the modulation of inflammatory responses. In particular, the regulation of ethanol-metabolizing enzymes and suppression of reactive oxygen species (ROS) generation may play key roles in mitigating alcohol-induced liver injury [7].

Despite these promising biological activities, the therapeutic efficacy and underlying mechanisms of Laennec’s effect in alcoholic fatty liver disease remain insufficiently characterized. Current treatment strategies for ALD, including corticosteroid therapy in severe alcoholic hepatitis, provide only limited short-term benefits and are associated with significant adverse effects, highlighting the need for alternative therapeutic approaches.

Therefore, the present study aimed to evaluate the hepatoprotective effects of Laennec in an experimental model of alcohol-induced fatty liver and to elucidate its potential mechanisms of action, with a focus on oxidative stress, inflammatory responses, and alcohol-metabolizing enzyme systems.

## 2. Materials and Methods

### 2.1. Chemicals and Reagents

Laennec injectable products (lot No. 0111042) were obtained from GC Green Cross Wellbeing (Seoul, Republic of Korea). Laennec is a human placental extract containing various amino acids, including cysteine, glutamic acid, and glycine, which are known precursors of glutathione. Lieber–DeCarli Regular Liquid Diet for Rodents (control, #710027) and Ethanol Lieber–DeCarli Regular Liquid Diet for Rodents (#710260) were purchased from Dyets Inc. (Bethlehem, PA, USA). Alcohol dehydrogenase (ADH) and aldehyde dehydrogenase (ALDH) activity assay kits were obtained from BioVision Inc. (Milpitas, CA, USA). All other reagents used in this study were of analytical grade.

### 2.2. Animal and Housing Conditions

In this experiment, seven-week-old male Sprague Dawley rats were purchased from OrientBio (Seongnam, Republic of Korea). The rats were housed in a semi-specific-pathogen-free (SPF) facility at the College of Medicine, Hanyang University, under controlled environmental conditions (23 ± 2 °C, 60 ± 5% relative humidity, and a 12 h light/dark cycle). The rats were housed two per cage (MSRSII cages, OrientBio) with free access to food and water. All animals were acclimatized for 7 days prior to the experiment.

### 2.3. Experimental Design and Treatment

The present study was designed to evaluate the efficacy of Laennec against alcohol-induced fatty liver and to investigate its mechanism of action. A total experimental period of 6 weeks was employed. Fatty liver was induced by administering an ethanol-containing liquid diet for 2 weeks. Subsequently, animals continued to receive the ethanol diet for an additional 4 weeks, during which treatment agents were intravenously administered.

After 4 weeks of alcohol exposure (including the induction period), one animal from the ethanol-fed group was sacrificed to confirm hepatic steatosis histologically. The animals were randomly assigned to four groups (n = 5 per group): (i) control group (CTL), receiving a control diet and physiological saline (intravenous, i.v.); (ii) ethanol group (ET), receiving an ethanol diet and physiological saline (i.v.); (iii) low-dose group (LD), receiving an ethanol diet and Laennec injection (0.41 mL/kg, i.v., twice weekly); and (iv) high-dose group (HD), receiving an ethanol diet and Laennec injection (1.03 mL/kg, i.v., twice weekly).

### 2.4. Diet Preparation

Liquid diets were prepared according to the Lieber–DeCarli method. The control diet was prepared by dissolving 221.78 g of powder in 840 mL of water to obtain 1 L of solution. The ethanol diet was prepared by mixing 132.28 g of powder with 821 mL of water and 67 mL of ethanol to yield 1 L of diet.

### 2.5. Biochemical and Molecular Analyses

The body weights were recorded weekly throughout the experimental period. Blood samples (2 mL) were collected from the tail vein for biochemical analysis. Serum liver function parameters, including alanine aminotransferase (ALT), aspartate aminotransferase (AST), AST/ALT ratio, total bilirubin, and alkaline phosphatase (ALP), were measured. Inflammatory cytokines, including interleukin-6 (IL-6) and tumor necrosis factor-α (TNF-α), were analyzed using the serum samples. In addition, liver tissue was analyzed for the expression of signal transducer and activator of transcription 3 (MyBioSource, Seoul, Republic of Korea, Cat No.: MBS7279224), glutathione peroxidase (GPx, Abcam, Hanam, Republic of Korea, Cat No.: ab102530), and catalase (Abcam, Cat No.: ab83464) to evaluate the oxidative stress-related pathways. Hepatic alcohol metabolism was assessed by measuring CYP2E1(CYP2E1 ELISA Kit, FineTest, Seongnam, Republic of Korea, ER0402), ADH and ALDH activities using commercial colorimetric assay kits, according to the manufacturer’s instructions(ADH Assay Kit, Abcam, Cat no: ab102533 and ALDH Assay Kit, Abcam, Cat No.: ab155893).

### 2.6. Histopathological Analysis

At the end of the experimental period, the rats were sacrificed, and liver tissues were excised for histopathological examination. Liver samples were photographed, and portions were either fixed in 10% neutral-buffered formalin or snap-frozen in liquid nitrogen and stored at −80 °C. Formalin-fixed tissues were embedded in paraffin, sectioned into 5 μm-thick slices, and stained with hematoxylin and eosin (H&E). Histopathological changes were examined using a light microscope (Olympus BH-2, Tokyo, Japan). The inflammatory cell infiltration was evaluated at 200× magnification and scored as follows: 0 (no inflammation), 1 (≤2 foci), 2 (2–4 foci), and 3 (≥4 foci). Quantitative image analysis was performed using NIS-Elements BR software Ver. 4.00 (Nikon, Tokyo, Japan). Steatosis was quantified using a standardized threshold-based image analysis in NIS-Elements, calculating the percentage of the steatotic area relative to the total tissue area using the formula Steatosis Area (%) = (Steatotic Area/Total Tissue Area) × 100.

### 2.7. Statistical Analysis

All data are presented as the mean ± standard deviation (SD). Statistical analyses were performed using IBM SPSS Statistics (version 29.0.2.0; IBM Corp., Armonk, NY, USA) and GraphPad Prism 10.1.1 (GraphPad Software, San Diego, CA, USA). Normality and homogeneity of variance were assessed using the Shapiro–Wilk test and Levene’s test, respectively. Depending on the results, one-way ANOVA, Welch’s ANOVA, or the non-parametric Kruskal–Wallis test was applied. Appropriate post hoc tests (Tukey’s HSD, Dunnett’s, Games–Howell, or Dunn’s test) were used. Statistical significance was set at *p* < 0.05.

### 2.8. Ethical Approval

All animal experimental procedures were approved by the Institutional Animal Care and Use Committee (IACUC) of Hanyang University (Approval No. HY-IACUC-25-0279) and were conducted in accordance with the relevant guidelines and regulations.

## 3. Results

### 3.1. Changes in Body Weight and Food Intake

**Figure 1:**
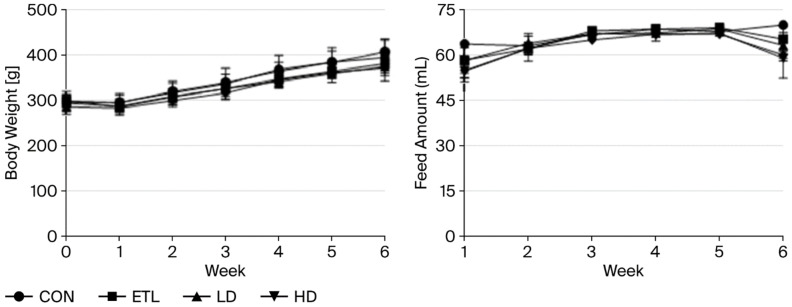
Effects of Laennec on body weight gain and food intake. Data are expressed as the mean ± SD (*n* = 5). No significant differences were observed between groups.

No significant differences in body weight gain or food intake were observed between the experimental groups throughout the study period (Figure 1).

### 3.2. Gross Morphological Findings of the Liver

**Figure 2:**
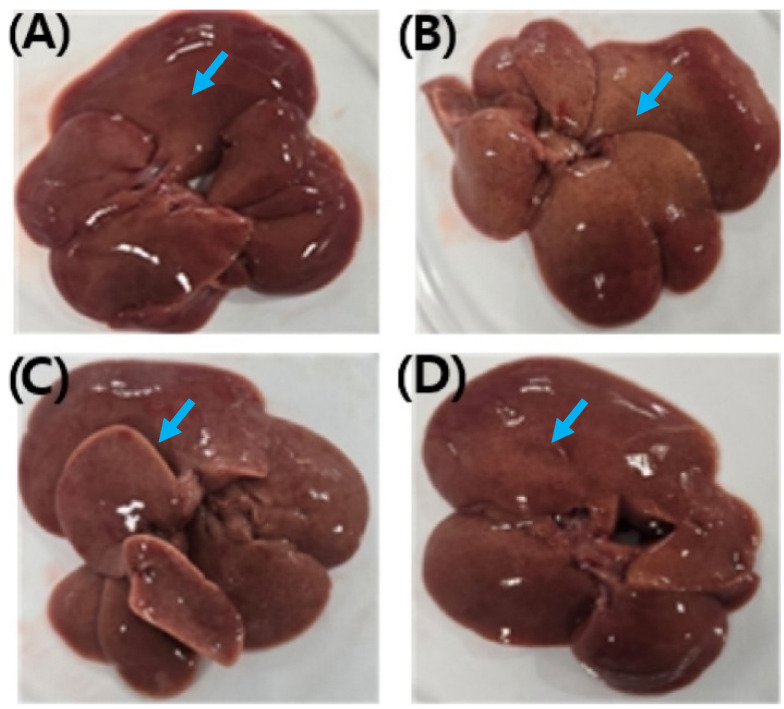
Representative gross morphology of liver tissues. The ethanol-treated group shows pale discoloration and irregular surface morphology (arrow) compared with the control group. The Laennec-treated groups exhibit partial improvement. (**A**) Control; (**B**) ETL (ethanol-treated); (**C**) LD (low dose); (**D**) HD (high dose).

At necropsy, livers from the ethanol-treated group exhibited a paler coloration and irregular surface morphology compared with the control group. In contrast, the Laennec-treated groups showed a partial improvement in liver appearance, with reduced surface irregularity and color similar to control regardless of the dose (Figure 2).

### 3.3. Serum Hepatic Enzyme Profiles

**Figure 3:**
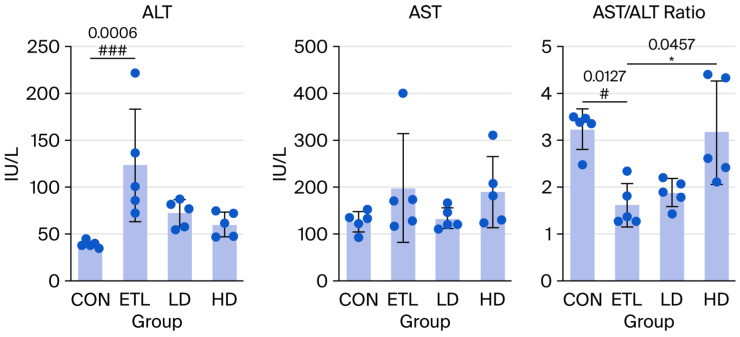
Effects of Laennec on serum hepatic enzyme levels (ALT, AST, and AST/ALT ratio). Data are expressed as the mean ± SD (*n* = 5). ### *p* < 0.01 vs. control; *, # *p* < 0.05 vs. ethanol-treated group. ALT, alanine aminotransferase; AST, aspartate aminotransferase.

The serum ALT levels were significantly elevated in the ethanol-treated group compared with the control group (*p* < 0.01). Laennec treatment reduced the ALT levels in a dose-dependent manner; however, these reductions were not statistically significant. The AST levels showed an increasing trend in the ethanol-treated group but did not reach statistical significance. The Laennec-treated groups exhibited a decreasing trend in AST levels. The AST/ALT ratio was significantly reduced in the ethanol-treated group (*p* < 0.05), indicating ethanol-induced hepatic inflammation. This ratio was significantly restored in the high-dose Laennec group (*p* < 0.05) (Figure 3).

### 3.4. Hepatic Steatosis and Inflammation Scores

**Figure 4:**
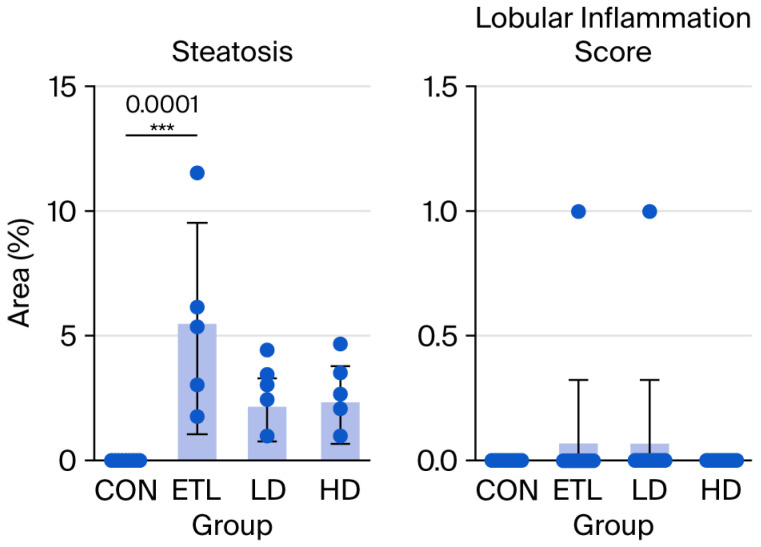
Effects of Laennec on hepatic steatosis and inflammation scores. Data are expressed as the mean ± SD (*n* = 5). *** *p* < 0.05 vs. ethanol-treated group.

Histological scoring revealed a significant increase in hepatic lipid accumulation in the ethanol-treated group compared with the controls. Laennec administration reduced hepatic steatosis in both treatment groups, with the most pronounced effect observed in the high-dose group. The hepatic inflammation scores were elevated following ethanol exposure and showed a decreasing trend with Laennec treatment; however, these changes were not statistically significant (Figure 4).

### 3.5. Effects on Alcohol-Metabolizing Enzymes (ADH, ALDH, and CYP2E1)

**Figure 5:**
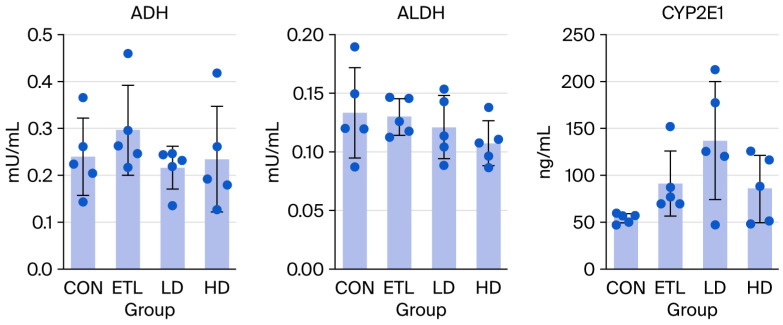
Effects of Laennec on alcohol-metabolizing enzymes (ADH, ALDH, and CYP2E1). Data are expressed as the mean ± SD (*n* = 5). ADH, alcohol dehydrogenase; ALDH, aldehyde dehydrogenase; CYP2E1, cytochrome P450 2E1.

Ethanol exposure increased the ADH activity, which was attenuated by Laennec treatment. No significant differences in ALDH activity were observed between the groups. CYP2E1 activity was suppressed in a dose-dependent manner following Laennec administration compared with the ethanol-treated group (Figure 5).

### 3.6. Antioxidant Enzyme Activity and STAT3 Phosphorylation

**Figure 6:**
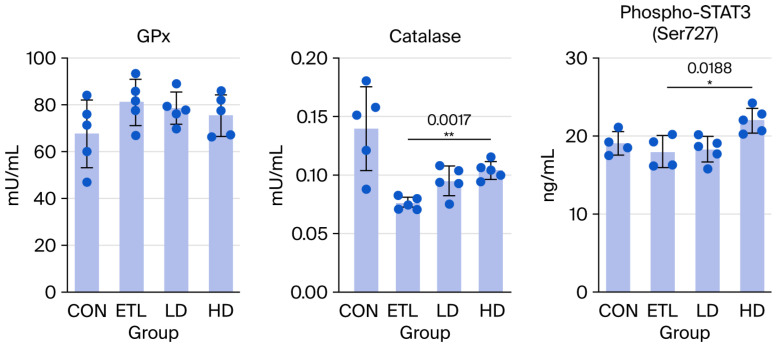
Effects of Laennec on antioxidant enzyme activity (GPx, catalase) and STAT3 (Ser727) phosphorylation. Data are expressed as the mean ± SD (*n* = 5). * *p* < 0.05 vs. alcohol-treated group. ** *p* < 0.05 vs. alcohol-treated group. GPx, glutathione peroxidase.

The GPx activity showed no significant differences between groups. The catalase activity, which was reduced in the ethanol-treated group, was significantly restored following Laennec administration, particularly in the high-dose group (*p* < 0.05). Phosphorylation of STAT3 (Ser727) was decreased by ethanol exposure and significantly restored by Laennec treatment, with the most pronounced effect observed at the high dose (*p* < 0.05) (Figure 6).

### 3.7. Serum Inflammatory Cytokines (IL-6 and TNF-α)

**Figure 7:**
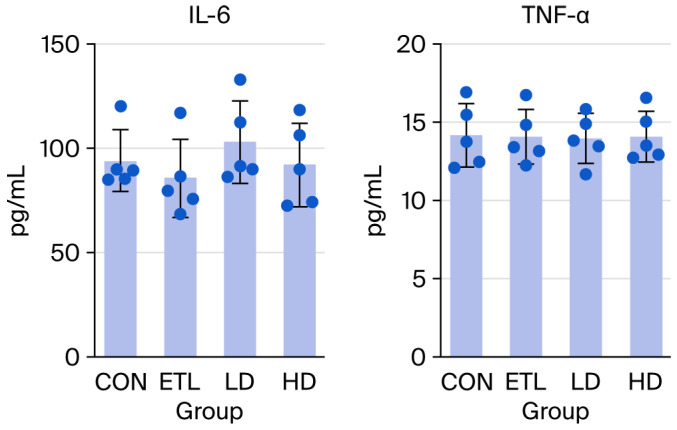
Effects of Laennec on serum inflammatory cytokines (IL-6 and TNF-α). Data are expressed as the mean ± SD (*n* = 5). No significant differences were observed between the groups. IL-6, interleukin-6; TNF-α, tumor necrosis factor-alpha.

No significant differences in the serum IL-6 and TNF-α levels were observed between the ethanol- and Laennec-treated groups (Figure 7).

### 3.8. Histopathological Findings (H&E Staining)

**Figure 8:**
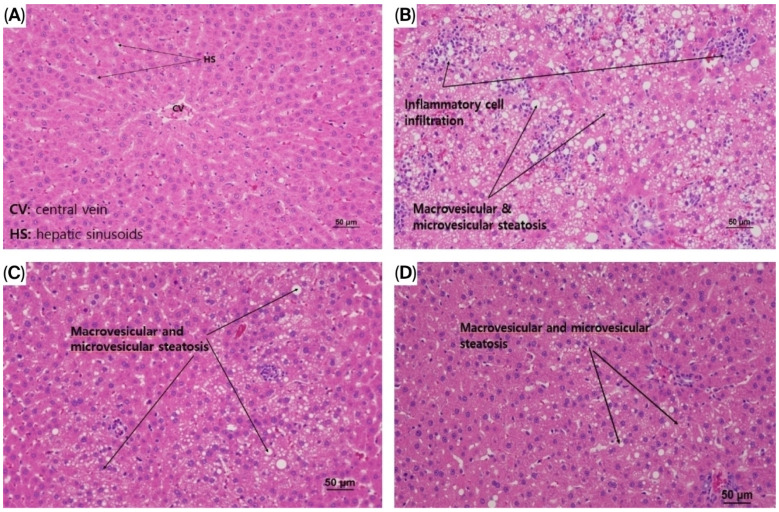
Representative histopathological images of liver tissues stained with hematoxylin and eosin (H&E). Sections were fixed in 10% neutral-buffered formalin, embedded in paraffin, and sectioned at 4–5 μm. Scale bar = 100 μm; magnification, ×200. (**A**) Control (normal); (**B**) ETL (ethanol-treated); (**C**) LD (low dose); (**D**) HD (high dose).

The histopathological analysis of liver tissues stained with hematoxylin and eosin (H&E) revealed preserved hepatic architecture in the control group, characterized by intact central veins (CV) and well-organized hepatic sinusoids. In contrast, the ethanol-treated group exhibited marked hepatic steatosis, as evidenced by extensive lipid droplet accumulation, along with prominent inflammatory cell infiltration. The Laennec-treated groups showed attenuation of these pathological alterations, including reduced lipid droplet accumulation and decreased inflammatory infiltration, with the most pronounced improvement observed in the high-dose group (Figure 8).

## 4. Discussion

Laennec is a human placental hydrolysate composed primarily of water-soluble bioactive components (112 mg/2 mL), produced using stringent safety and viral inactivation processes, including enzymatic hydrolysis, high-temperature sterilization, and nucleic acid testing to ensure product safety [8]. In South Korea, a comparable formulation (Laennec) has undergone clinical evaluation, demonstrating non-inferior efficacy in improving serum alanine aminotransferase (ALT) levels in patients with alcoholic and non-alcoholic fatty liver disease, with a low incidence of adverse drug reactions [9]. Previous studies have reported that human placental hydrolysates exert hepatoprotective effects in models of drug-induced liver injury [10]. In particular, in acetaminophen-induced liver injury models, placental hydrolysate treatment attenuated hepatocellular necrosis and improved liver function markers through the modulation of phase I/II detoxification enzymes, enhancement of antioxidant defenses, and regulation of inflammatory responses [11]. These findings suggest that placental hydrolysates may exert multi-target protective effects by enhancing hepatic detoxification capacity and reducing oxidative stress. Alcoholic liver disease (ALD) is characterized by hepatic steatosis, oxidative stress, and inflammation resulting from chronic alcohol exposure [12]. Ethanol metabolism is primarily mediated by alcohol dehydrogenase (ADH) and aldehyde dehydrogenase (ALDH), while cytochrome P450 2E1 (CYP2E1) becomes increasingly important during chronic alcohol consumption [13]. Induction of CYP2E1 leads to the excessive generation of reactive oxygen species (ROS), promoting lipid peroxidation, mitochondrial dysfunction, and hepatocellular injury [14]. In addition, acetaldehyde, a toxic intermediate of ethanol metabolism, forms adducts with proteins, lipids, and nucleic acids, further exacerbating cellular damage [15]. In the present study, alcohol administration induced significant hepatic injury, as evidenced by increased ALT levels, hepatic lipid accumulation, and histopathological alterations. Laennec treatment attenuated these changes, particularly at higher doses. Notably, Laennec suppressed CYP2E1 activity in a dose-dependent manner, suggesting reduced ROS generation during ethanol metabolism. Furthermore, the restoration of catalase activity and STAT3 (Ser727) phosphorylation indicates the partial recovery of antioxidant defenses and signaling pathways associated with hepatocellular protection and regeneration. Inflammation is a central driver of ALD progression. Activation of Kupffer cells by endotoxins and damage-associated molecular patterns promotes the release of pro-inflammatory cytokines, including tumor necrosis factor-α (TNF-α) and interleukin-6 (IL-6), which contribute to hepatocyte injury and disease progression [16]. Although no significant changes in the circulating cytokine levels were observed in this study, the improvement in histological inflammatory infiltration suggests that Laennec may exert localized anti-inflammatory effects within hepatic tissue. Despite advances in understanding ALD pathogenesis, effective pharmacological treatments remain limited. Current therapies, such as corticosteroids, provide modest short-term benefits but are associated with significant adverse effects and limited long-term efficacy [17]. Therefore, there is increasing interest in alternative therapeutic strategies targeting oxidative stress, inflammation, and metabolic dysregulation. Biologically derived compounds, including placental hydrolysates, may offer therapeutic potential due to their multi-mechanistic actions. However, this study has several limitations. First, the experimental duration may not fully reflect chronic alcohol exposure in humans. Second, the mechanistic evaluation was limited to selected biochemical and signaling markers. Future studies using long-term models and comprehensive molecular analyses are warranted to further elucidate the mechanisms underlying the hepatoprotective effects of Laennec.

## 5. Conclusions

Laennec administration significantly mitigated alcohol-induced hepatocellular injury and steatosis, as evidenced by reduced serum ALT levels, an improved AST/ALT ratio, the attenuation of hepatic lipid accumulation and inflammation, and favorable histopathological changes. These effects were dose-dependent and most pronounced at the high dose. Mechanistically, Laennec suppressed CYP2E1 activity and modulated alcohol-metabolizing enzymes without affecting ALDH while partially restoring antioxidant defenses through increased catalase activity and STAT3 (Ser727) phosphorylation. Collectively, these findings indicate that Laennec exerts potent hepatoprotective effects against alcohol-induced liver injury.

## Data Availability

No new data were created or analyzed in this study. Data sharing is not applicable to this article.

## Abbreviations

The following abbreviations are used in this manuscript:

ALD	Alcoholic liver disease
ADH	Alcohol dehydrogenase
ALDH	Aldehyde dehydrogenase
CYP2E1	Cytochrome P450 2E1
TNF-α	Tumor necrosis factor-α
IL-6	Interleukin-6
